# Thrombolysis in Myocardial Infarction Frame Count for Coronary Blood Flow Evaluation during Interventional Diagnostic Procedures

**DOI:** 10.3390/medicina59122185

**Published:** 2023-12-15

**Authors:** Tatsuro Yamazaki, Yuichi Saito, Hideki Kitahara, Yoshio Kobayashi

**Affiliations:** Department of Cardiovascular Medicine, Chiba University Graduate School of Medicine, 1-8-1, Inohana, Chuo-ku, Chiba 260-8670, Japan; tyamazaki1990@gmail.com (T.Y.); hidekita.0306@gmail.com (H.K.); yuiryosuke@msn.com (Y.K.)

**Keywords:** interventional diagnostic procedure, Thrombolysis in Myocardial Infarction frame count, acetylcholine provocation test

## Abstract

*Background and Objectives*: An interventional diagnostic procedure (IDP), including intracoronary acetylcholine (ACh) provocation and coronary physiological testing, is recommended as an invasive diagnostic standard for patients suspected of ischemia with no obstructive coronary arteries (INOCA). Recent guidelines suggest Thrombolysis In Myocardial Infarction frame count (TFC) as an alternative to wire-based coronary physiological indices for diagnosing coronary microvascular dysfunction. We evaluated trajectories of TFC during IDP and the impact of ACh provocation on TFC. *Materials and Methods*: This was a single-center, retrospective study. Patients who underwent IDP to diagnose INOCA were included and divided into two groups according to the positive or negative ACh provocation test. Wire-based invasive physiological assessment was preceded by ACh provocation tests and intracoronary isosorbide dinitrate (ISDN). We evaluated TFC at three different time points during IDP; pre-ACh, post-ISDN, and post-hyperemia. *Results*: Of 104 patients, 58 (55.8%) had positive ACh provocation test. In the positive ACh group, resting mean transit time (Tmn) and baseline resistance index were significantly higher than in the negative ACh group. Post-ISDN TFC was significantly correlated with resting Tmn (r = 0.31, *p* = 0.002). Absolute TFC values were highest at pre-ACh, followed by post-ISDN and post-hyperemia in both groups. All between-time point differences in TFC were statistically significant in both groups, except for the change from pre-ACh to post-ISDN in the positive ACh group. *Conclusions*: In patients suspected of INOCA, TFC was modestly correlated with Tmn, a surrogate of coronary blood flow. The positive ACh provocation test influenced coronary blood flow assessment during IDP.

## 1. Introduction

Ischemic heart disease is a leading cause of mortality and morbidity across the world. Epicardial coronary artery disease (CAD) is a representative subtype of ischemic heart disease, and coronary angiography has been believed to be an invasive standard for CAD evaluation. However, previous studies reported that less than half of patients have epicardial CAD on coronary angiography when angina is suspected [1,2]. For example, among 398,978 patients with suspected CAD undergoing coronary angiography, no epicardial CAD, defined as <20% stenosis in all vessels, was reported in approximately 40% [1]. In this context, ischemia with no obstructive coronary arteries (INOCA) may play significant roles in ischemic heart disease, with widespread clinical interest recently [3,4,5]. Because INOCA is reportedly associated with increased cardiovascular event risk and impaired quality of life [6,7,8], accurate diagnosis and subsequent therapeutic strategies in patients with INOCA are clinically important, among which vasospastic angina (VSA) and coronary microvascular dysfunction (CMD) are major subtypes. VSA is a clinical manifestation of myocardial ischemia induced by dynamic epicardial coronary obstruction caused by vasoreactivity dysfunction. In 1959, Prinzmetal first reported the clinical and electrocardiographic manifestations (i.e., transient ST-segment elevation) of a disorder that may be associated with epicardial coronary artery spasm [3,4,5]. Subsequently, other types of vasomotor disorders with chest symptoms accompanied by transient ST-segment depression or T-wave inversion have been reported. These clinical entities caused by epicardial coronary spasms were grouped as VSA. CMD is a part of the criteria for microvascular angina, which is a clinical manifestation of myocardial ischemia with no epicardial CAD. In this clinical entity, myocardial ischemia is caused by structural remodeling of coronary microvasculature or vasomotor disorders affecting the coronary arterioles. Although underlying mechanisms remain unclear, epicardial VSA can co-exist with microvascular angina, which is associated with a worse prognosis [3,4,5]. In addition to lifestyle modification, VSA can be treated with calcium channel blockers, nitrates, and nicorandil, and patients with microvascular angina typically receive β-blockers, calcium channel blockers, ranolazine, angiotensin-converting enzyme inhibitors, and others. 

Recent international guidelines recommend an interventional diagnostic procedure (IDP), namely the invasive evaluation of VSA and CMD with intracoronary acetylcholine (ACh) provocation and coronary physiological testing, in patients suspected of INOCA [3,4,5]. Invasive diagnostic strategies, using coronary angiography and IDP can be performed in the catheterization laboratory to differentiate between VSA, microvascular angina, and non-cardiac chest symptoms. Nonetheless, the protocol of IDP has not been established yet, particularly in the sequential order and coronary physiological metrics during the invasive testing. For instance, although either ACh provocation or coronary physiological testing is performed during IDP, a preceding ACh provocation test may influence on following physiological assessment, hypothesizing that coronary blood flow significantly changes during IDP [9,10]. In addition, guidelines suggest Thrombolysis in Myocardial Infarction frame count (TFC) on coronary angiography as a readily available alternative to wire-based coronary physiological indices for CMD evaluation [4], such as coronary flow reserve (CFR) and index of microcirculatory resistance (IMR), while the impact of IDP on TFC is uncertain. In the present study, we evaluated trajectories of TFC during IDP and the impact of the ACh provocation test on TFC. 

## 2. Materials and Methods

### 2.1. Study Population

This was a single-center, retrospective study at Chiba University Hospital in Japan. A total of 126 patients suspected of INOCA underwent IDP with both ACh provocation test and wire-based invasive coronary physiological assessment between December 2015 and July 2023, among whom patients with significant epicardial CAD de-fined as fractional flow reserve (FFR) ≤0.80 (n = 9), no assessable coronary angiograms for TFC (n = 4), missing physiological data (n = 4), non-elective IDP such as acute coronary syndrome (n = 2), maintenance hemodialysis (n = 2), and significant coronary-pulmonary artery fistulas (n = 1) were excluded. Thus, 104 patients were included in the present analysis. Cardiovascular risk factors such as hypertension, diabetes, dyslipidemia, and current smoking were defined according to the Japanese Association of Cardiovascular Intervention and Therapeutics criteria [11]. A blood examination was performed on admission. Hypertension was defined as a previous diagnosis of hypertension or previous antihypertensive medications, or newly diagnosed hypertension during hospitalization with systolic blood pressure ≥ 140 mm Hg and/or diastolic blood pressure ≥ 90 mm Hg. Diabetes was defined as a previous diagnosis of diabetes or previous glucose-lowering medications, or hemoglobin A1c ≥ 6.5% on admission. Dyslipidemia was defined as having low-density lipoprotein cholesterol ≥ 140 mg/dL, high-density lipoprotein cholesterol < 40 mg/dL, or fasting triglycerides > 150 mg/dL, or a previous diagnosis of dyslipidemia. Low- and high-density lipoprotein cholesterol levels were evaluated in either a fasting or non-fasting condition. Other laboratory data including hemoglobin and creatinine were also assessed. Additionally, patients with a history of smoking within the past year were defined as being current smokers. An estimated glomerular filtration rate <60 mL/min/1.73 m^2^ was defined as chronic kidney disease. This study was done in accordance with the Declaration of Helsinki. The ethics committee of Chiba University Graduate School of Medicine approved this study (M10348/27 July 2022). Informed consent was ascertained in the form of opt-out.

### 2.2. Acetylcholine Provocation Test

**Figure 1:**
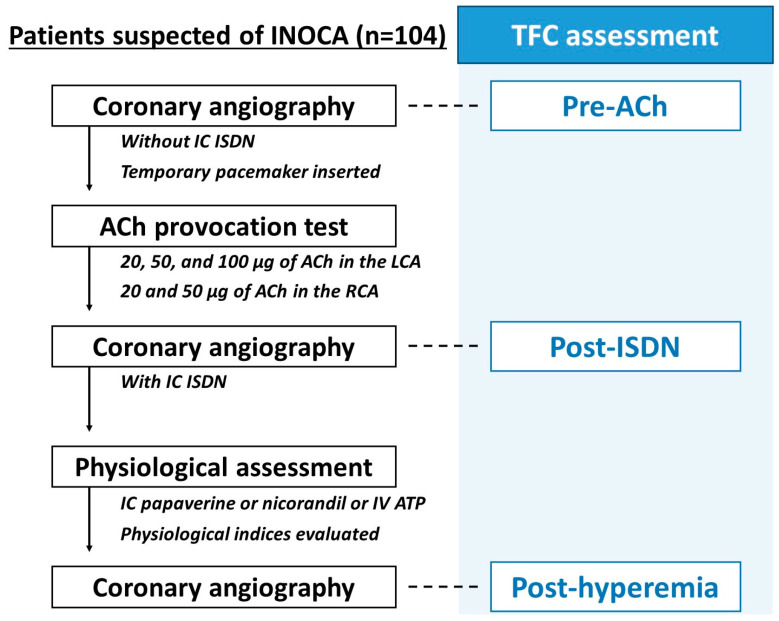
Flow of IDP and timing of the TFC assessment. After performing control coronary angiography without intracoronary administration of ISDN as a reference angiogram, the ACh provocation test was performed in LCA and RCA. Following ACh provocation testing, 1 mg of ISDN was administered to achieve epicardial coronary vasodilation, irrespective of the ACh rest results, and a coronary angiogram with intracoronary ISDN was obtained. After that, a coronary physiological assessment was done in the LAD by the bolus-saline injection thermodilution method using a pressure-temperature sensor guidewire (PressureWire Certus and PressureWire X; Abbott Vascular, Santa Clara, USA). We evaluated TFC at three different time points during IDP; pre-ACh, post-ISDN, and post-hyperemia. ACh, acetylcholine; IDP, interventional diagnostic procedure; ISDN, isosorbide dinitrate; LAD, left anterior descending coronary artery; LCA, left coronary artery; RCA, right coronary artery; TFC, Thrombolysis in Myocardial Infarction frame count; Tmn, mean transit time; TIMI, Thrombolysis in Myocardial Infarction.

Intracoronary ACh provocation tests and wire-based coronary physiological assessments were performed during the same catheterization procedure. Invasive physio-logical assessment was preceded by ACh provocation tests in this study (Figure 1). Intracoronary ACh provocation tests were performed according to the recent Japanese guidelines [4,12], as previously reported [13]. In brief, vasodilation drugs, such as calcium channel blockers, long-acting nitrates, and nicorandil, were discontinued at least 48 h before the provocation tests. Coronary angiography was performed via radial, brachial, or femoral artery with a 4 to 6 Fr catheter per local standards. After administration of unfractionated heparin, operators conducted baseline (control) coronary angiography without intracoronary administration of nitrates (isosorbide dinitrate [ISDN]) as a reference angiogram, followed by temporary pacemaker insertion in the right ventricle via brachial, femoral, or internal jugular vein. Intracoronary ACh was administered at incremental doses of 20, 50, and 100 μg into the left coronary artery initially, and 20 and 50 μg into the right coronary artery subsequently. Coronary angiography was performed to evaluate angiographic vasospasm after 60 s from the start of each ACh injection. Following ACh provocation testing, 1 mg of ISDN was administered to achieve epicardial coronary vasodilation, irrespective of the ACh rest results. Angiographically significant coronary artery spasm was defined as total or subtotal occlusion induced by intracoronary ACh provocation. Ischemic electrocardiographic changes suggestive of the presence of myocardial ischemia included ST-segment depression or elevation ≥0.1 mV in at least two contiguous leads [4,12]. Positive ACh provocation test was defined as significant angiographic epicardial vasospasm in the left anterior descending coronary artery (LAD) accompanied by ischemic chest symptoms and/or electrocardiographic change [4,12]. In this study, patients were divided into the positive and negative ACh test groups. 

### 2.3. Coronary Physiological Assessment

After ACh provocation tests and coronary angiography on intracoronary administration of ISDN, coronary physiological indices were invasively measured [10,14]. The coronary physiological assessment was done in the LAD by the bolus-saline injection thermodilution method using a pressure-temperature sensor guidewire (PressureWire Certus and PressureWire X; Abbott Vascular, Santa Clara, CA, USA) [15]. Six Fr guiding catheter without side holes were used, and a pressure wire was inserted into the distal third of the LAD after equalization at the ostium of the left coronary artery. Mean aortic pressure (Pa) and mean distal coronary pressure (Pd) were measured, and 3 ml of room-temperature saline was injected three times from a guiding catheter into the LAD. Mean transit time (Tmn) was automatically calculated with a dedicated system (CoroFlow system, Coroventis Research, Uppsala, Sweden). Maximum hyperemia was archived using intracoronary administration of papaverine (12 mg) nicorandil (2 mg), or intravenous adenosine triphosphate (140 μg/kg/min) through the central vein [9,14]. Pa, Pd, and Tmn were measured at resting and hyperemic conditions. In the present study, a ratio of resting Pd to Pa at a resting condition (resting Pd/Pa), FFR, baseline resistance index (BRI), IMR, and CFR were calculated with resting and hyperemic Pa, Pd, and Tmn. In addition, resistive reserve ratio (RRR) and microvascular resistance reserve (MRR), novel coronary physiological parameters evaluating coronary vascular dilation capacity, were also calculated [16,17,18,19,20]. In the original methods, MRR is invasively measured by the continuous-saline injection thermodilution method using a dedicated microcatheter, namely the RayFlow catheter (Hexacath, Rueil-Malmaison, France) [18]. However, in the present study, MRR was calculated with physiological indices measured by the bolus-thermodilution method, rather than measured by absolute coronary blood flow using a continuous-thermodilution method. The formulae of these indices were as follows: resting Pd/Pa = resting Pd/resting Pa; FFR = hyperemic Pd/hyperemic Pa; BRI = resting Pd × resting Tmn; IMR = hyperemic Pd × hyperemic Tmn; CFR = resting Tmn/hyperemic Tmn; RRR = BRI/IMR = (resting Pd × resting Tmn)/(hyperemic Pd × hyperemic Tmn) = CFR × (resting Pd/hyperemic Pd); and MRR = (resting Pa × resting Tmn)/(hyperemic Pd × hyperemic Tmn) = CFR × (resting Pa/hyperemic Pd) = (CFR/FFR) × (resting Pa/hyperemic Pa) = RRR × (resting Pa/resting Pd), as briefly summarized in our previous reports [10,20]. In this study, the definitions of abnormal CFR and IMR were CFR < 2.5 and IMR ≥ 25, respectively, and the patients with abnormal CFR and/or IMR (i.e., CFR < 2.5 and/or IMR ≥ 25) were defined as having CMD [3,4,5,6,21]. 

### 2.4. Thrombolysis in Myocardial Infarction Frame Count

The concept of TFC was originally proposed as an objectively quantitative method to assess coronary blood [22]. In this study, TFC was evaluated in the LAD as previously reported [22,23]. Briefly, the first frame was the frame where the contrast medium first fully enters the target coronary artery. This occurred when three criteria were met as follows: First, fully or near fully concentrated contrast medium extended across the entire width of the ostium of the artery lumen; second, dye reached both borders of the artery; finally, dye moved ahead. The last frame is the frame when the dye first enters the distal landmark branch [22]. For LAD, the distal landmark branch was the distal bi-furcation, which was described as the “mustache”, “pitchfork”, or “whale’s tail” in the original report [22]. The number of frames was counted for contrast to transit between the ostium and distal landmark of the LAD. If the left circumflex artery was selectively engaged, the TFC began when the dye first touched both borders at the ostium of the LAD [22]. TFC assessment was performed with an angiogram which well-visualized the entire LAD and the distal landmarks. If these landmarks were not well-visualized, another visible landmark that was close to these landmarks was chosen. Because coronary angiography images were acquired at 15 frames/s in our institution, the number of frame counts was multiplied by two to adjust a frame rate as described in the original report (i.e., 30 frames/s) [22]. Additionally, the value was divided by 1.7 to correct the longer length of LAD than the right coronary and left circumflex arteries [22]. In the present study, TFC was measured at three different time points during IDP as shown in Figure 1. First, a coronary angiogram on control angiography was used to measure TFC as “pre-ACh”. Second, “post-ISDN” TFC was assessed with a coronary angiogram archived from coronary angiography with intracoronary administration of ISDN. Finally, after coronary physiological assessment, final coronary angiography was performed to confirm whether there were any complications or not. “Post-hyperemia” TFC was measured from that final angiogram. Post-ISDN TFC was evaluated on coronary angiography immediately after intracoronary nitrates, while post-hyperemia TFC was assessed a few minutes after the induction of hyperemia. In this study, TFC was analyzed by two experienced cardiologists who were blinded to patient characteristics and physiological findings.

### 2.5. Endpoints and Statistical Analysis

The main interest of the present study was to evaluate changes in TFC between the positive and the negative ACh groups during IDP. Coronary physiological indices were also compared between the two groups. Contentious variables were expressed as mean ± standard deviation and compared using a Student *t*-test. Paired *t*-test was conducted in the analyses of TFC value between each different point during IDP. Categorical variables were represented as n (%) and analyzed with Fisher’s exact test. The relation between post-ISDN TFC and resting Tmn was assessed with Pearson’s correlation coefficient. All statistical analyses were performed using JMP pro version 16.0 (SAS Institute Inc., Cary, NC, USA). A value of *p* < 0.05 was considered statistically significant.

## 3. Results

Of 104 patients, 58 (55.8%) had positive ACh provocation tests. Baseline characteristics are summarized in Table 1. Although the rate of current smoking was significantly higher in the positive ACh group than in the negative ACh group, baseline characteristics were overall similar between the two groups. 

Table 2 shows the findings of ACh provocation and coronary physiological tests. The number of patients who had ischemic chest symptoms or significant electrocardiographic change was significantly higher in the positive ACh group than in the negative ACh group (Table 2). The rate of hyperemic agents used in this study was not significantly different between the positive and negative groups (Table 2). In the positive ACh group, resting Tmn and BRI were significantly higher than in the negative ACh group (Table 2). Although not statistically significant, CFR, RRR, and MRR tended to be higher in the positive rather than negative ACh groups. The rate of CMD was numerically lower in the positive ACh group (24.1% vs. 34.8%, *p* = 0.28) (Table 2). 

**Figure 2:**
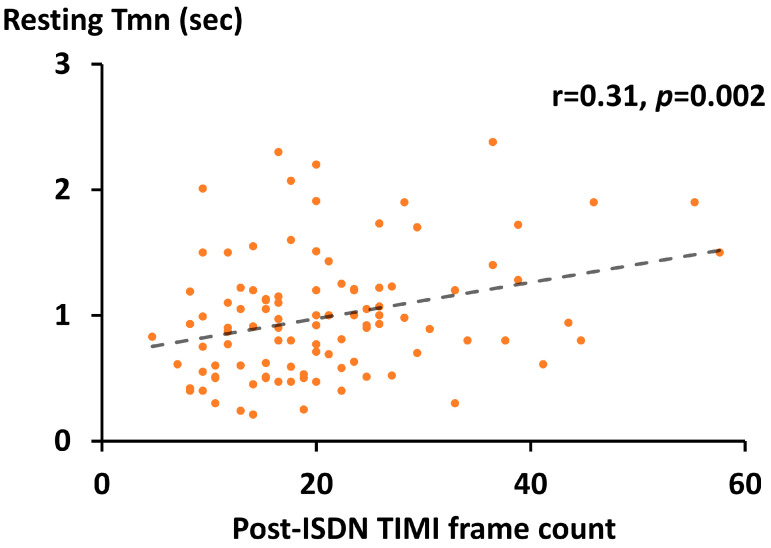
Relation between post-ISDN TFC and resting Tmn. Scatterplots (orange) with a regression line (dash line), ISDN, isosorbide dinitrate; TFC, Thrombolysis in Myocardial Infarction frame count; Tmn, mean transit time; TIMI, Thrombolysis in Myocardial Infarction.

As shown in Figure 1, TFC was evaluated at three time points, pre-ACh, post-ISDN, and post-hyperemia. The mean time interval from the induction of maximum hyperemia to final coronary angiography (i.e., post-hyperemia) was 2.5 ± 1.7 min. The post-ISDN TFC was significantly and positively correlated with resting Tmn (r = 0.31, *p* = 0.002) (Figure 2). 

**Figure 3:**
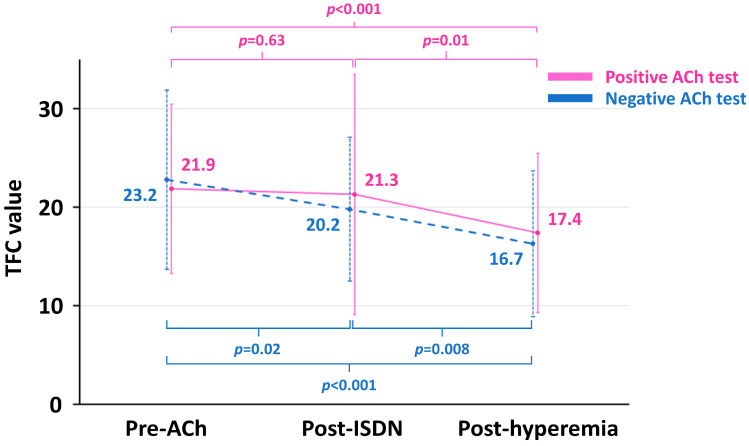
Trajectories of TFC value in the positive and negative ACh groups. ACh, acetylcholine; ISDN, isosorbide dinitrate; TFC, Thrombolysis in Myocardial Infarction frame count.

Absolute TFC values were highest at pre-ACh, followed by post-ISDN and post-hyperemia in both positive and negative ACh groups (Figure 3). All between-time point differences in TFC were statistically significant in both groups, except for the change from pre-ACh to post-ISDN in the positive ACh group (Figure 3). Absolute TFC values at each point did not differ significantly between the two groups (Table 2).

## 4. Discussion

The present study showed trajectories of TFC during IDP and confirmed a significant but modest correlation between post-ISDN TFC and resting Tmn, both of which represent coronary blood flow. Overall, TFC values decreased from pre-ACh to post-ISDN and post-hyperemia during IDP. In patients with positive ACh provocation test, however, TFC values did not change significantly from pre-ACh to post-ISDN, suggesting that a preceding ACh provocation test influenced on following physiological assessment even after administration of intracoronary nitrates. 

Recent guidelines recommend IDP for the diagnosis of INOCA [3,4,5], because accurate diagnosis of VSA and CMD, both of which are major etiologies of INOCA, can lead to appropriate therapeutic strategies and improved patient outcomes [24]. However, the protocol of IDP has been unestablished and is a matter of debate across the world. For instance, the sequential order of ACh provocation and coronary physiological tests during IDP differs between countries and regions. In the European consensus document, a preceding physiological assessment on intracoronary nitrates and hyperemic agents followed by ACh provocation is recommended [25], while the opposite sequence of IDP is widely performed in Japan [4,9,10]. From a European perspective, the preceding ACh tests may result in an inaccurate assessment of physiological testing potentially because of the alteration of coronary circulation by ACh administration [25,26]. From a Japanese perspective, on the other hand, the preceding coronary physiological testing using vasodilating drugs including intracoronary nitrates and hyperemic agents may result in the underdiagnosis of VSA [4,27]. Both sequential orders of IDP in European countries and Japan include strengths and limitations in their procedures. 

In this context, we evaluated the trajectories of TFC during IDP. In the original report, TFC was proposed as an objectively quantitative method to estimate coronary blood flow in patients with acute myocardial infarction [22]. Currently, the increase in TFC is suggested as a surrogate of the presence of CMD in cases without significant epicardial CAD [4]. In previous studies using intracoronary Doppler wire, average peak velocity was modestly and significantly correlated with TFC, with a correlation coefficient ranging from −0.58 to −0.32 [23,28,29], which is in line with our results. Despite being a surrogate, TFC is a simple and readily available method to estimate coronary blood flow in clinical practice [22]. Overall, TFC values decreased from pre-ACh (control) to post-ISDN and post-hyperemia during IDP, indicating that coronary blood flow became faster after intracoronary nitrates and maximal hyperemia. Abaci et al. demonstrated that intracoronary administration of nitrates significantly increased TFC values from 26.4 ± 11.9 to 32.8 ± 13.3 (*p* < 0.001), along with elevated heart rate and reduced blood pressure [30]. On the other hand, another study showed considerably decreased TFC values from baseline to post-nitroglycerine administration (38.1 ± 10.1 to 19.3 ± 8.4), despite the lack of statistical comparisons [31]. Although uncertain, these conflicting results of TFC changes after intracoronary nitrates may be due to the included patient populations. While the former study by Abaci et al. mainly enrolled patients with stable angina, those included in the later study had coronary slow flow phenomenon [31]. Thus, the decrease in TFC thorough vasodilation response might be archived by nitrate infusion, particularly in patients with reduced coronary blood flow at baseline. Interestingly, however, TFC values were unchanged in the positive ACh group when intra-coronary ISDN was administrated in the present study. We previously reported that the patients with a positive result of the ACh provocation test had significantly higher resting Tmn values compared with those without (1.18 ± 0.51 vs. 0.75 ± 0.31 s, *p* < 0.001) [10], as well as other previous studies [9,32,33]. In addition, a recent European multi-center study evaluating the diagnostic ability of the “ACh rechallenge” to detect coexisting microvascular spasm among patients with epicardial spasm showed that micro-vascular spasm remained after intracoronary administration of ISDN during ACh provocation test in approximately 70% of study patients [34]. These results suggest that decreased post-ISDN coronary blood flow induced by ACh injection was not fully restored by intracoronary administration of nitrates possibly due to microvascular spasm by ACh provocation and insufficient microvascular dilation response by ISDN administration. Although the absolute post-ISDN TFC value was not significantly different between the two groups, resting Tmn was significantly higher in the positive ACh group, potentially resulting in numerically higher values of CFR, RRR, and MRR in the positive rather than negative ACh groups in this study. In terms of the relation between maximum hyperemia and a change in TFC, a Doppler wire study showed that TFC values decreased from baseline to hyperemia (20.9 ± 10.4 vs. 11.3 ± 8.0, *p* < 0.001) [28]. Because the induction of maximum hyperemia decreases coronary microvascular resistance and increases coronary blood flow, it is reasonable that hyperemic agents reduce a TFC value. 

Taken together, the present study showed a decrease in absolute TFC values from pre-ACh to post-ISDN and post-hyperemia during IDP, suggesting that coronary blood flow was increased by intracoronary nitrates and hyperemic agents. In addition, the preceding ACh provocation test influenced the results of the following coronary physiological assessment, including wire-based indices and TFC. These findings may be important for interventional cardiologists when performing IDP. Future studies are warranted to define the protocol of IDP, especially in the sequential order of ACh provocation and coronary physiological tests during the diagnostic procedure. 

This study had some limitations. This was a single-center study done retrospectively. The sample size was relatively small. Noninvasive stress tests for evaluating the presence of myocardial ischemia were not performed uniformly. The selection of hyperemic agents was left to the operator’s discretion. Although the different characteristics of each hyperemic agent (i.e., intracoronary papa-verine and nicorandil and intravenous adenosine and adenosine triphosphate) in safety, efficacy, and availability have been reported in previous studies, the rates of hyper-emic agents used in this study were similar between the two groups. The positive ACh test in this study was determined only in the LAD. Final coronary angiography was not necessarily performed immediately after the injection of the hyperemic agent, although the time interval from archiving maximum hyperemia to post-hyperemia was overall short. Because wire-based physiological assessment was conducted in the LAD, TFC was evaluated in the same coronary artery. In this study, MRR was assessed by using a bolus-saline thermodilution method rather than using a continuous-saline thermodilution method [18].

## 5. Conclusions

In patients suspected of INOCA, TFC was modestly correlated with Tmn, both of which are surrogates of coronary blood flow. Although TFC decreased from pre-ACh to post-ISDN and post-hyperemia during IDP in most cases, no significant change in TFC by intracoronary nitrate was observed in patients with positive ACh provocation test. The results of the preceding ACh provocation test influenced the following coronary physiological assessment, suggesting that further debates are needed to standardize and optimize the protocol of IDP.

## Figures and Tables

**Table 1:** Baseline characteristics.

Variable	All (n = 104)	Positive ACh (n = 58)	Negative ACh (n = 46)	*p* Value
Age (years)	64.3 ± 12.1	63.8 ± 11.0	64.9 ± 13.4	0.63
Men	54 (51.9%)	33 (56.9%)	21 (45.7%)	0.32
Body mass index (kg/m^2^)	24.2 ± 3.8	24.4 ± 3.8	23.9 ± 3.9	0.49
Hypertension	57 (54.8%)	31 (53.5%)	26 (56.5%)	0.84
Diabetes	15 (14.4%)	8 (13.8%)	7 (15.2%)	1.00
Dyslipidemia	78 (75.0%)	48 (82.8%)	30 (65.2%)	0.07
Current smoking	21 (20.2%)	16 (27.6%)	5 (10.9%)	0.049
Chronic kidney disease	22 (21.2%)	12 (20.7%)	10 (21.7%)	1.00
Previous MI	6 (5.8%)	5 (8.6%)	1 (2.2%)	0.22
Hemoglobin (g/dL)	13.6 ± 1.6	13.8 ± 1.6	13.4 ± 1.6	0.31
LDL cholesterol (mg/dL)	113 ± 34	112 ± 37	116 ± 30	0.55
HDL cholesterol (mg/dL)	63 ± 18	61 ± 15	67 ± 22	0.11
Medical treatment				
Antiplatelet	34 (32.7%)	21 (36.2%)	13 (28.3%)	0.41
Statin	49 (47.1%)	31 (53.5%)	18 (39.1%)	0.17
β-blocker	18 (17.3%)	10 (17.2%)	8 (17.4%)	1.00
ACE-i or ARB	29 (27.9%)	18 (31.0%)	11 (23.9%)	0.51
Calcium channel blocker	52 (50.0%)	32 (55.2%)	20 (43.5%)	0.32
Nitrate	20 (19.2%)	14 (24.1%)	6 (13.0%)	0.21

ACE-i, angiotensin-converting enzyme inhibitor; ACh, acetylcholine; ARB, angiotensin II receptor blocker; HDL, high-density lipoprotein; LDL, low-density lipoprotein; MI, myocardial infarction.

**Table 2:** Findings of ACh provocation, coronary physiological tests, and TFC.

	All (n = 104)	Positive ACh (n = 58)	Negative ACh (n = 46)	*p* Value
ACh provocation test				
Chest symptom	73 (70.2%)	54 (93.1%)	19 (41.3%)	<0.001
ECG change	59 (56.7%)	49 (84.5%)	10 (21.7%)	<0.001
Hyperemic agent				0.85
Intracoronary papaverine	47 (45.2%)	25 (43.1%)	22 (47.8%)	
Intracoronary nicorandil	37 (35.6%)	22 (37.9%)	15 (32.6%)	
Intravenous ATP	20 (19.2%)	11 (19.0%)	9 (19.6%)	
Physiological findings				
Resting Pd/Pa	0.95 ± 0.02	0.95 ± 0.02	0.95 ± 0.02	0.27
FFR	0.91 ± 0.04	0.91 ± 0.04	0.92 ± 0.04	0.20
Resting Tmn (s)	0.99 ± 0.49	1.12 ± 0.51	0.81 ± 0.40	<0.001
Hyperemic Tmn (s)	0.23 ± 0.12	0.25 ± 0.13	0.21 ± 0.11	0.10
BRI	90.4 ± 44.7	101.7 ± 43.9	76.2 ± 42.0	0.003
IMR	18.1 ± 10.3	19.4 ± 10.8	16.4 ± 9.4	0.14
CFR	4.9 ± 2.7	5.2 ± 2.8	4.5 ± 2.5	0.16
RRR	5.7 ± 3.1	6.1 ± 3.3	5.1 ± 2.8	0.11
MRR	6.1 ± 3.3	6.6 ± 3.5	5.4 ± 2.9	0.08
CMD (CFR < 2.5 and/or IMR ≥ 25)	30 (28.9%)	14 (24.1%)	16 (34.8%)	0.28
TFC value				
Pre-ACh	22.5 ± 8.8	21.9 ± 8.6	23.2 ± 9.1	0.44
Post-ISDN	20.8 ± 10.3	21.3 ± 12.2	20.2 ± 7.3	0.57
Post-hyperemia	17.1 ± 7.8	17.4 ± 8.1	16.7 ± 7.4	0.64

ACh, acetylcholine; ATP, adenosine triphosphate; BRI, baseline resistance index; CFR, coronary flow reserve; CMD, coronary microvascular dysfunction; ECG, electrocardiography; FFR, fractional flow reserve; IMR, index of microcirculatory resistance; ISDN, isosorbide dinitrate; MRR, microvascular resistance reserve; Pa, mean aortic pressure; Pd, mean distal coronary pressure; Pd/Pa, ratio of distal coronary pressure to aortic pressure; RRR, resistive reserve ratio; TFC, Thrombolysis In Myocardial Infarction frame count; Tmn, mean transit time.

## Data Availability

The data of this study are available upon reasonable request.

## References

[1] Patel M.R., Peterson E.D., Dai D., Brennan J.M., Redberg R.F., Anderson H.V., Brindis R.G., Douglas P.S. (2010). Low diagnostic yield of elective coronary angiography. N. Engl. J. Med..

[2] Reeh J., Therming C.B., Heitmann M., Højberg S., Sørum C., Bech J., Husum D., Dominguez H., Sehestedt T., Hermann T. (2019). Prediction of obstructive coronary artery disease and prognosis in patients with suspected stable angina. Eur. Heart J..

[3] Knuuti J., Wijns W., Saraste A., Capodanno D., Barbato E., Funck-Brentano C., Prescott E., Storey R.F., Deaton C., Cuisset T. (2020). 2019 ESC Guidelines for the diagnosis and management of chronic coronary syndromes. Eur. Heart J..

[4] Hokimoto S., Kaikita K., Yasuda S., Tsujita K., Ishihara M., Matoba T., Matsuzawa Y., Mitsutake Y., Mitani Y., Murohara T. (2023). JCS/CVIT/JCC 2023 Guideline Focused Update on Diagnosis and Treatment of Vasospastic Angina (Coronary Spastic Angina) and Coronary Microvascular Dysfunction. Circ. J..

[5] Virani S.S., Newby L.K., Arnold S.V., Bittner V., Brewer L.C., Demeter S.H., Dixon D.L., Fearon W.F., Hess B., Johnson H.M. (2023). 2023 AHA/ACC/ACCP/ASPC/NLA/PCNA Guideline for the Management of Patients with Chronic Coronary Disease: A Report of the American Heart Association/American College of Cardiology Joint Committee on Clinical Practice Guidelines. Circulation.

[6] Shimokawa H., Suda A., Takahashi J., Berry C., Camici P.G., Crea F., Escaned J., Ford T., Yii E., Kaski J.C. (2021). Clinical characteristics and prognosis of patients with microvascular angina: An international and prospective cohort study by the Coronary Vasomotor Disorders International Study (COVADIS) Group. Eur. Heart J..

[7] Schumann C.L., Mathew R.C., Dean J.L., Yang Y., Balfour P.C., Shaw P.W., Robinson A.A., Salerno M., Kramer C.M., Bourque J.M. (2021). Functional and Economic Impact of INOCA and Influence of Coronary Microvascular Dysfunction. JACC Cardiovasc. Imaging.

[8] Gulati M., Khan N., George M., Berry C., Chieffo A., Camici P.G., Crea F., Kaski J.C., Marzilli M., Merz C.N.B. (2023). Ischemia with no obstructive coronary artery disease (INOCA): A patient self-report quality of life survey from INOCA international. Int. J. Cardiol..

[9] Saito Y., Nishi T., Kato K., Kitahara H., Kobayashi Y. (2022). Resistive reserve ratio and microvascular resistance reserve in patients with coronary vasospastic angina. Heart Vessel..

[10] Yamazaki T., Saito Y., Yamashita D., Kitahara H., Kobayashi Y. (2023). Impact of preceding acetylcholine provocation testing on following coronary physiological assessment during an interventional diagnostic procedure. J. Cardiol..

[11] Ando H., Yamaji K., Kohsaka S., Ishii H., Wada H., Yamada S., Sawano M., Inohara T., Numasawa Y., Ikari Y. (2022). Japanese Nationwide PCI (J-PCI) Registry Annual Report 2019: Patient demographics and in-hospital outcomes. Cardiovasc. Interv. Ther..

[12] Suzuki S., Kaikita K., Yamamoto E., Jinnouchi H., Tsujita K. (2021). Role of acetylcholine spasm provocation test as a pathophysiological assessment in nonobstructive coronary artery disease. Cardiovasc. Interv. Ther..

[13] Saito Y., Saito Y., Kato K., Kobayashi Y. (2022). Gender differences in factors associated with vasospastic angina. Int. J. Cardiol..

[14] Kawase Y., Matsuo H., Kuramitsu S., Shiono Y., Akasaka T., Tanaka N., Amano T., Kozuma K., Nakamura M., Yokoi H. (2022). Clinical use of physiological lesion assessment using pressure guidewires: An expert consensus document of the Japanese association of cardiovascular intervention and therapeutics-update 2022. Cardiovasc. Interv. Ther..

[15] Yamazaki T., Nishi T., Saito Y., Tateishi K., Kato K., Kitahara H., Kobayashi Y. (2022). Discrepancy between plaque vulnerability and functional severity of angiographically intermediate coronary artery lesions. Cardiovasc. Interv. Ther..

[16] Lee S.H., Lee J.M., Park J., Choi K.H., Hwang D., Doh J.H., Nam C.W., Shin E.S., Hoshino M., Murai T. (2020). Prognostic Implications of Resistive Reserve Ratio in Patients With Coronary Artery Disease. J. Am. Heart Assoc..

[17] Toya T., Ahmad A., Corban M.T., Özcan I., Sara J.D., Sebaali F., Escaned J., Lerman L.O., Lerman A. (2021). Risk Stratification of Patients With NonObstructive Coronary Artery Disease Using Resistive Reserve Ratio. J. Am. Heart Assoc..

[18] De B.B., Pijls N.H.J., Gallinoro E., Candreva A., Fournier S., Keulards D.C.J., Sonck J., Van’t V.M., Barbato E., Bartunek J. (2021). Microvascular Resistance Reserve for Assessment of Coronary Microvascular Function: JACC Technology Corner. J. Am. Coll. Cardiol..

[19] Boerhout C.K.M., Lee J.M., de Waard G.A., Mejia-Renteria H., Lee S.H., Jung J.H., Hoshino M., Echavarria-Pinto M., Meuwissen M., Matsuo H. (2023). Microvascular resistance reserve: Diagnostic and prognostic performance in the ILIAS registry. Eur. Heart J..

[20] Yamazaki T., Saito Y., Yamashita D., Kitahara H., Kobayashi Y. (2023). Factors Associated with Impaired Resistive Reserve Ratio and Microvascular Resistance Reserve. Diagnostics.

[21] Demir O.M., Boerhout C.K.M., de Waard G.A., van de Hoef T.P., Patel N., Beijk M.A.M., Williams R., Rahman H., Everaars H., Kharbanda R.K. (2022). Comparison of Doppler Flow Velocity and Thermodilution Derived Indexes of Coronary Physiology. JACC Cardiovasc. Interv..

[22] Gibson C.M., Cannon C.P., Daley W.L., Dodge J.T., Alexander B., Marble S.J., McCabe C.H., Raymond L., Fortin T., Poole W.K. (1996). TIMI frame count: A quantitative method of assessing coronary artery flow. Circulation.

[23] Dutta U., Sinha A., Demir O.M., Ellis H., Rahman H., Perera D. (2023). Coronary Slow Flow Is Not Diagnostic of Microvascular Dysfunction in Patients with Angina and Unobstructed Coronary Arteries. J. Am. Heart Assoc..

[24] Ford T.J., Stanley B., Good R., Rocchiccioli P., McEntegart M., Watkins S., Eteiba H., Shaukat A., Lindsay M., Robertson K. (2018). Stratified Medical Therapy Using Invasive Coronary Function Testing in Angina: The CorMicA Trial. J. Am. Coll. Cardiol..

[25] Kunadian V., Chieffo A., Camici P.G., Berry C., Escaned J., Maas A.H.E.M., Prescott E., Karam N., Appelman Y., Fraccaro C. (2020). An EAPCI Expert Consensus Document on Ischaemia with Non-Obstructive Coronary Arteries in Collaboration with European Society of Cardiology Working Group on Coronary Pathophysiology & Microcirculation Endorsed by Coronary Vasomotor Disorders International Study Group. Eur. Heart J..

[26] Ford T.J., Berry C. (2019). How to Diagnose and Manage Angina Without Obstructive Coronary Artery Disease: Lessons from the British Heart Foundation CorMicA Trial. Interv. Cardiol..

[27] Beck S., Pereyra V.M., Seitz A., McChord J., Hubert A., Bekeredjian R., Sechtem U., Ong P. (2021). Invasive Diagnosis of Coronary Functional Disorders Causing Angina Pectoris. Eur. Cardiol..

[28] Manginas A., Gatzov P., Chasikidis C., Voudris V., Pavlides G., Cokkinos D.V. (1999). Estimation of coronary flow reserve using the Thrombolysis In Myocardial Infarction (TIMI) frame count method. Am. J. Cardiol..

[29] Tanedo J.S., Kelly R.F., Marquez M., Burns D.E., Klein L.W., Costanzo M.R., Parrillo J.E., Hollenberg S.M. (2001). Assessing coronary blood flow dynamics with the TIMI frame count method: Comparison with simultaneous intracoronary Doppler and ultrasound. Catheter. Cardiovasc. Interv..

[30] Abaci A., Oguzhan A., Eryol N.K., Ergin A. (1999). Effect of potential confounding factors on the thrombolysis in myocardial infarction (TIMI) trial frame count and its reproducibility. Circulation.

[31] Xu X., Zhou J., Zhang Y., Li Q., Guo L., Mao Y., He L. (2022). Evaluate the Correlation between the TIMI Frame Count, IMR, and CFR in Coronary Microvascular Disease. J. Interv. Cardiol..

[32] Yamanaga K., Tsujita K., Komura N., Kaikita K., Sakamoto K., Miyazaki T., Saito M., Ishii M., Tabata N., Akasaka T. (2015). Single-wire pressure and flow velocity measurement for quantifying microvascular dysfunction in patients with coronary vasospastic angina. Am. J. Physiol. Heart. Circ. Physiol..

[33] Takagi A., Arai K., Hosaka M., Komatsu Y., Gunnji K., Tanimoto K., Ishizuka N., Tsurumi Y., Hagiwara N. (2008). Noninvasive prediction of angiographic spasm provocation using trans-thoracic Doppler echocardiography in patients with coronary spastic angina. Circ. J..

[34] Seitz A., Feenstra R., Konst R.E., Martínez Pereyra V., Beck S., Beijk M., van de Hoef T., van Royen N., Bekeredjian R., Sechtem U. (2022). Acetylcholine Rechallenge: A First Step Toward Tailored Treatment in Patients with Coronary Artery Spasm. JACC Cardiovasc. Interv..

